# Post-Feeding Larval Mobility and Burial Behaviour of two Forensically Relevant Species, *Peckia* (*Peckia*) *chrysostoma* (Wiedemann) and *Peckia* (*Sarcodexia*) *lambens* (Wiedemann) (Diptera: Sarcophagidae)

**DOI:** 10.1007/s13744-026-01363-0

**Published:** 2026-02-21

**Authors:** Henrique Rafael Pontes Ferreira, Taciano Moura Barbosa, Simão Dias Vasconcelos

**Affiliations:** Lab of Insects of Forensic Importance, Dept of Zoology, Univ Federal de Pernambuco, Recife, Pernambuco, Brazil

**Keywords:** Forensic entomology, Flesh fly, Larval dispersal, Experimental arena

## Abstract

In forensic entomology (FE), understanding larval dispersal is crucial for determining key data such as the time, distance, trajectory, speed, and burial depth for pupation. This study aimed to characterize the mobility, horizontal dispersal and burial behaviour of larvae of two forensically important species of Sarcophagidae: *Peckia* (*Peckia*) *chrysostoma* (Wiedemann) and *Peckia* (*Sarcodexia*) *lambens* (Wiedemann). To achieve that, we designed and assessed the efficacy of a Larval Mobility Arena (LMA). Third-instar larvae of each species were released in the centre of the LMA and the following variables were quantified: speed, trajectory orientation, distance travelled, time to reach the soil, and time to burial. *Peckia* (*P*.) *chrysostoma* showed a higher speed (x̄ = 1.58) (*P* = 0.002) and a greater total distance travelled (x̄ = 61.58) (*P* = 0.04) compared to *P*. (*S*.) *lambens* (speed x̄ = 1.31; total distance travelled x̄ = 53.21). Mobility in *Peckia* (*P*.) *chrysostoma* was oriented, with displacements concentrated towards specific directions in the arena, whereas *P.* (*S*.) *lambens* displayed a scattered dispersal. The larvae moved fast and quickly reached the substrate for pupation, but the time spent in the arena was longer for *P.* (*S*.) *lambens* (x̄ = 84.12) (*P* < 0.001). Larvae of both species immediately buried themselves once they encountered the substrate, with no differences in burrowing time (*P* = 0.68). These findings improve our understanding of larval behaviour and its applications in forensic entomology, supporting experts in locating larvae, inferring parameters of dispersal distances, and improving the accuracy of minimum post-mortem interval estimation.

**Supplementary Information:**

The online version contains supplementary material available at 10.1007/s13744-026-01363-0.

## Introduction

Post-feeding dispersal and burial are remarkable evolutionary traits found in most necrophagous insect dipteran larvae, which allow them to escape from predators and pathogens present in the necrobiome. Typically, necrophagous species of Calliphoridae and Sarcophagidae undergo three instars during which the larvae feed as much as possible until resources are exhausted or until they accumulate enough energy to complete their life cycle (Byrd and Tomberlin 2019). Because the profound tissue and organ reorganization associated with metamorphosis requires stable and suitable conditions, late third-instar larvae actively leave the feeding substrate and search for an appropriate pupation site in a process known as post-feeding larval dispersal (Greenberg 1990; Brown et al. 2015).

Moving away from the decomposing matter increases their likelihood of survival, as necromass attracts a variety of scavengers, pathogens, parasitoids, predators, cannibalism, competing species, and others (Faria et al. 2004; Reigada and Godoy 2005). Dispersing enhances the probability of successful metamorphic transition after achieving developmental size thresholds (Levot et al. 1979; Ribeiro and Von Zuben 2010). Larval mobility may not be uniform, and each species exhibits a distinct pattern of horizontal dispersal characterized by speed, body contractions, and distance traversed (Greenberg 1990; Jales et al. 2023).

To ensure survival and protection, larvae of necrophagous Diptera bury themselves (Frederickx et al. 2014). However, burial must be superficial and, preferably, in soft soil, to allow breathing and the vertical ascension of the adult (Balme et al. 2012). Although some studies suggest that larvae can burrow up to 50 cm into the soil (Vogt and Woodburn 1982), a depth threshold of less than 10 cm seems to apply for most Calliphoridae species (Greenberg 1990; Godoy et al. 1995; Sharma et al. 2021).

Sarcophagidae play a well-established role in the decomposition process and are the second most frequent family colonizing animal carcasses and human cadavers. Although the ecological niche exploited by sarcophagids is expected to result in dispersal and burial patterns similar to those observed in Calliphoridae, quantitative studies on larval behavior in this family remain virtually absent (Oliveira and Vasconcelos 2018; Vasconcelos et al. 2016, 2022; Meira et al. 2020; Barros et al. 2024).

In the context of forensic entomology , data on larval dispersal contribute to the understanding of the dynamics of cadaver colonization, as well as the factors that influence the time required for larvae to begin dispersal, the trajectory, speed, and distance travelled (Gomes et al. 2006; Gomes and Von Zuben 2005). Data on the depth of burial for pupation provide clues about the likelihood of colonization of buried cadavers. A recent study in Argentina revealed a high diversity of Diptera species as colonizers of a corpse found inside in a buried vehicle, including Calliphoridae, Piophilidae and Phoridae (Pereira et al. 2025), but no Sarcophagidae species were registered.

Given the increasing use of Diptera pupae as entomological evidence for the post-mortem interval minimum (min PMI), it is imperative to obtain quantitative data on dispersal and burial of species in the Neotropical Region, especially of neglected taxa, such as Sarcophagidae. Understanding these factors can aid in the forensic application of species, particularly *Peckia* (*Peckia*) *chrysostoma* (Wiedemann) and *Peckia* (*Sarcodexia*) *lambens* (Wiedemann), which have been documented as colonizers of human cadavers in Brazil (Vasconcelos et al. 2014; Barros et al. 2024).

In this study, we characterised the behaviour of necrophagous larvae at the post-feeding stage using an experimental arena under laboratory conditions, aiming to achieve the following objectives: i) quantify the horizontal dispersal in terms of direction (independent or gregarious), trajectory and speed of movement; ii) describe the pre-burial behaviour, such as the time elapsed until reaching the soil; iii) the spatial pattern of dispersal sites chosen by larvae in the arena; and iv) the time elapsed until complete burial.

We tested the following experimental hypotheses: 1) Mimicking real case scenarios, larvae disperse in a multidirectional pattern; 2) Larvae tend to exhibit a conspecific-induced behaviour, following the first dispersing individuals; 3) The speed of movement is higher in larger specimens; 4) The bigger the larvae the longer it will take until complete burial; and, lastly, 5) Because both species share the genus, there will be no difference in the overall patterns of mobility and burial between *P.* (*P.*) *chrysostoma* and *P.* (*S.*) *lambens*.

## Methods

### Insect Rearing

The insects used in the experiments were obtained from laboratory colonies reared for at least five generations under controlled conditions at a temperature of 25 ± 2°C, a relative humidity of 60% ± 10%, and a photoperiod of 12:12 (Light: Dark). Adults were maintained in 40 × 60 × 40 cm cages, supplied with a 50% sucrose solution in water *ad libitum*, and offered decomposing minced beef daily to promote ovarian development and stimulate larviposition. The females laid larvae, which were then transferred and kept in 250 mL plastic containers. Ground beef was provided as food (2 mg per larva), and vermiculite was used as a substrate for pupation (Barbosa et al. 2019; Ferreira et al. 2024).

To characterise the weight and length of the insects, we used a destructive subsample of larvae (*n* = 10) reared under the same conditions as those used in the experiment for each replicate. The larvae were individually washed to remove any adhering food and killed by immersion in warm water (3–5 s) at 50 ºC. Immediately afterwards, the body length was measured using a digital calliper with 0.1 mm precision, and the weight was recorded using an analytical balance (Tecnal®, 0.1 mg precision).

#### Larval Mobility Arena (LMA)

**Fig. 1:**
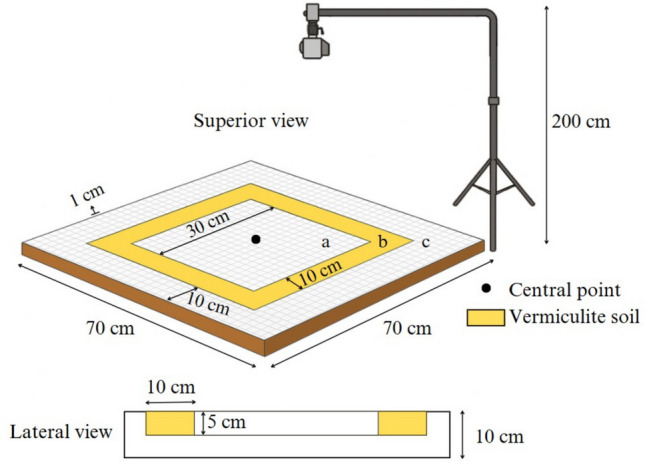
Scheme of the experimental Larval Mobility Arena (LMA): **a** central gridded area (30 cm^2^), **b** 10 cm-wide strips surrounding the central area, filled with vermiculite, and **c** additional gridded area, 10 cm wide

We designed an experimental arena called the Larval Mobility Arena (LMA), constructed using polystyrene (thickness: 50 mm), graph paper, acetate plastic, and vermiculite with a grain size of 3 mm. The LMA has a total area of 4,900 cm^2^, with the entire measurement section for larval movement covered with graph paper (markings: 1 mm) and a sheet of acetate plastic (thickness: 0.2 mm). The arena consisted of three parts: (a) a central, gridded area (30 cm^2^) where larvae are placed for behavioral observation; (b) a 10 cm-wide strip surrounding the central area, filled with vermiculite to simulate soil, with a depth of 5 cm to allow larval burial; and (c) an additional gridded area, 10 cm wide, providing a second opportunity for larvae to move even after finding a burial site (Fig. 1).

To eliminate any slope or elevation that could interfere with larval movement, all sections of the LMA were carefully levelled to ensure they remained perfectly aligned with the base floor. A filming system was installed at a height of 2 m above to capture the entire LMA within the camera frame, guaranteeing both image quality and consistency for subsequent video processing and behavioural analysis.

#### Larval Mobility

To assess the horizontal mobility of larvae at the post-feeding stage, we selected third-instar larvae of uniform age (approximately 72 h post-moulting) and size (about 19.6 mm for *P.* (*P.*) *chrysostoma*, 9.2 mm for *P.* (*S.*) *lambens*). For each species, a batch of 10 larvae was placed at the centre of the LMA using soft forceps, and this procedure was repeated ten times, resulting in ten repetitions per species. A one-minute acclimation period was allowed before the experiment to enable the larvae to settle, after which their behaviour was observed for five minutes.

To minimize external factors such as vibrations, odors, and ambient noise, the experiment was conducted in a closed room under controlled environmental conditions, including temperature (25 ± 2°C), relative humidity (60 ± 10%), and light intensity (100 lx). Larval mobility was characterised using quantitative variables: a) the total distance travelled by each larva (cm); b) the time spent until reaching the substrate; c) the average speed (cm/s); d) number of larvae buried itself upon contact with the substrate; e) the time elapsed from release in the LMA and encountering the substrate; and f) the proportion of larvae that surpassed the limits of the soil frame, continuing to disperse. Qualitative variables included: g) the trajectory of movement (i.e., the sum of spatial coordinates); and h) orientation of the larval movement (Table 1).

**Table 1:** Description of the variables observed in the Larval Mobility Arena (LMA), including measurement methods, units, and biological relevance

Variable	Description	Nature	Unit
Distance	Total distance travelled by the larva during the experiment	Quantitative	Millimetres (mm)
Mobility Time	Total time spent in movement by each lava	Quantitative	Seconds (s)
Speed	Travelled distance in a given time	Quantitative	Centimetres per second (cm/s)
Trajectory	Path followed by the larva, including orientation	Qualitative	Circular, linear, or curved
Orientation	Tendency of the larva to move consistently towards specific areas of the LMA, and whether it follows other larvae	Qualitative	Single direction (solitary) or grouped
Burrowing success rate	Proportion of larvae that became completely covered by the substrate and were no longer visible on the surface	Quantitative	Percentage (%)
Burrowing time	Time taken by the larvae to completely burrow in the substrate	Quantitative	Seconds (s)

#### Video Processing

Videos on larval behaviour were recorded using a digital camera (4 K quality, 30 fps, MP4 format) positioned 2 m above the LMA. This setup ensured that the entire LMA fitted within the frame without compromising image quality, providing a clear, high-resolution view of the millimetre paper.

Video recordings of each larva were analysed using Tracker® software (version 5.1.4). After importing the video into the software, the scale was set, and the origin point (y = 0, x = 0) was defined according to the central part of the LMA, with adjustments made for angle alignment.

The tracking of each larva was performed using a point on the cephalic region (anterior region) of the larva as a reference (Berrigan and Pepin 1995), evaluating the trajectory from the initial point at the origin of the LMA. Larval tracking began when the larva started moving, and coordinates were marked every 100 frames. Since the videos had approximately 10,000 frames, this approach ensured the best quality for analysing the experiments.

#### Data Analysis

The total distance travelled was obtained using the equation:

$$\text{d }= {\int }_{\mathrm{i}=1}^{\mathrm{n}}\sqrt{{({\mathrm{x}}_{\mathrm{i}}- {\mathrm{x}}_{\mathrm{i}-1})}^{2}+{({\mathrm{y}}_{\mathrm{i}}- {\mathrm{y}}_{\mathrm{i}-1})}^{2}}$$

where *d* is the total distance travelled by the larva (in mm, subsequently converted to cm), *x* and *y* represent the Cartesian coordinates at a given point (i). The velocity obtained for each sample considers the larva's displacement as uniform rectilinear motion, measured at each larval movement, using the measurement/position at each point (i) in the Cartesian plane from time (t) and position variations (y) of the subsequent (ti + 1 and yi + 1) and previous (ti-1 and yi-1) points, using the formula:

$${\mathrm{v}}_{\mathrm{y},\mathrm{i}} = \frac{{\mathrm{y}}_{\mathrm{i}+1}- {\mathrm{y}}_{\mathrm{i}-1}}{{\mathrm{t}}_{\mathrm{i}+1} - {\mathrm{t}}_{\mathrm{i}-1}}$$

The time spent by each larva in the LMA was measured by subtracting the initial time from the final time when the larva exited (in milliseconds, later converted to seconds). The same procedure was used to measure the time of burial upon encountering the vermiculite.

To examine the pattern of larval distance travelled (cm), a Generalized Linear Model (GLM) was fitted, based on the observed distance to predict the distance travelled based on the values of time (s) and speed (cm/s) for each species. The general formula of the model is given by:

$$Distance= \epsilon +\left(a\, x\, time\right)+(b\, x\, speed)$$

where ϵ = residual error, a and b = coefficients estimated by the model. The model was evaluated using RMSE (Root Mean Squared Error) and R^2^ (Coefficient of Determination).

Qualitative data were analysed by observing the movement path of each larva, and, for the sake of clarity, we classified the trajectory as either i) circular, ii) linear, or iii) curved. Another variable was the direction of movement, which could be individual or collective, with larvae potentially moving towards a common direction. To assess this, the location where the larva encountered the vermiculite was recorded by dividing the LMA into five quadrants, each measuring 10 cm on each side.

Quantitative data related to different species and variables were tested for normality using the Shapiro–Wilk test. Normally distributed data were analysed using ANOVA and Tukey's post hoc test. For non-normally distributed data, the Kruskal–Wallis test and Bonferroni's post hoc test were applied. All statistical analyses were performed using R® software (version 4.4.0) at a 5% significance level.

## Results

The insects were immediately active after release in the LMA, as most of the larvae left the centre of the LMA within 15 s. The average speed of movement was greater for *P.* (*P.*) *chrysostoma* than for *P.* (*S.*) *lambens* (χ^2^ = 9.2, df = 1, *P* = 0.002), and the mean total distance travelled was also longer for *P*. (*P*.) *chrysostoma* (χ^2^ = 3.89, df = 1, *P* = 0.04) (Table 2).

**Table 2:** Estimated time of larvae in the  Larval Mobility Arena (LMA), average speed, total distance travelled, time to burrow, along with weight and length data for *Peckia* (*P.*) *chrysostoma* and *Peckia* (*S.*) *lambens*. SD: Standard deviation

	*P. (P.) chrysostoma*			*P. (S.) lambens*		
	Mean (± SD)	Median	Range	Mean (± SD)	Median	Range
Weight (mg)	167.50 ± 6.80	168.2	159.63–176.91	98.60 ± 4.20	98.62	95.74–102.01
Length (mm)	19.55 ± 1.60	19.83	18.23–21.82	9.18 ± 0.60	9.23	8.56–9.54
Distance travelled by larvae (cm)	61.58 ± 27.82	58.46	25.43–149,0	53.21 ± 20.51	47.82	25.13–125.41
Time until reaching substrate (s)	52.58 ± 25.62	45,0	26.66–136.66	84.12 ± 36.05	80,0	41.66–263.33
Speed (cm/s)	1.58 ± 0.94	1.41	0.20–4.80	1.31 ± 0.82	1.26	0.11–3.56
Time for burial (s)	42.26 ± 33.37	36.20	5.66–167.90	40.91 ± 25.52	34.12	3.73–123.80

**Fig. 2:**
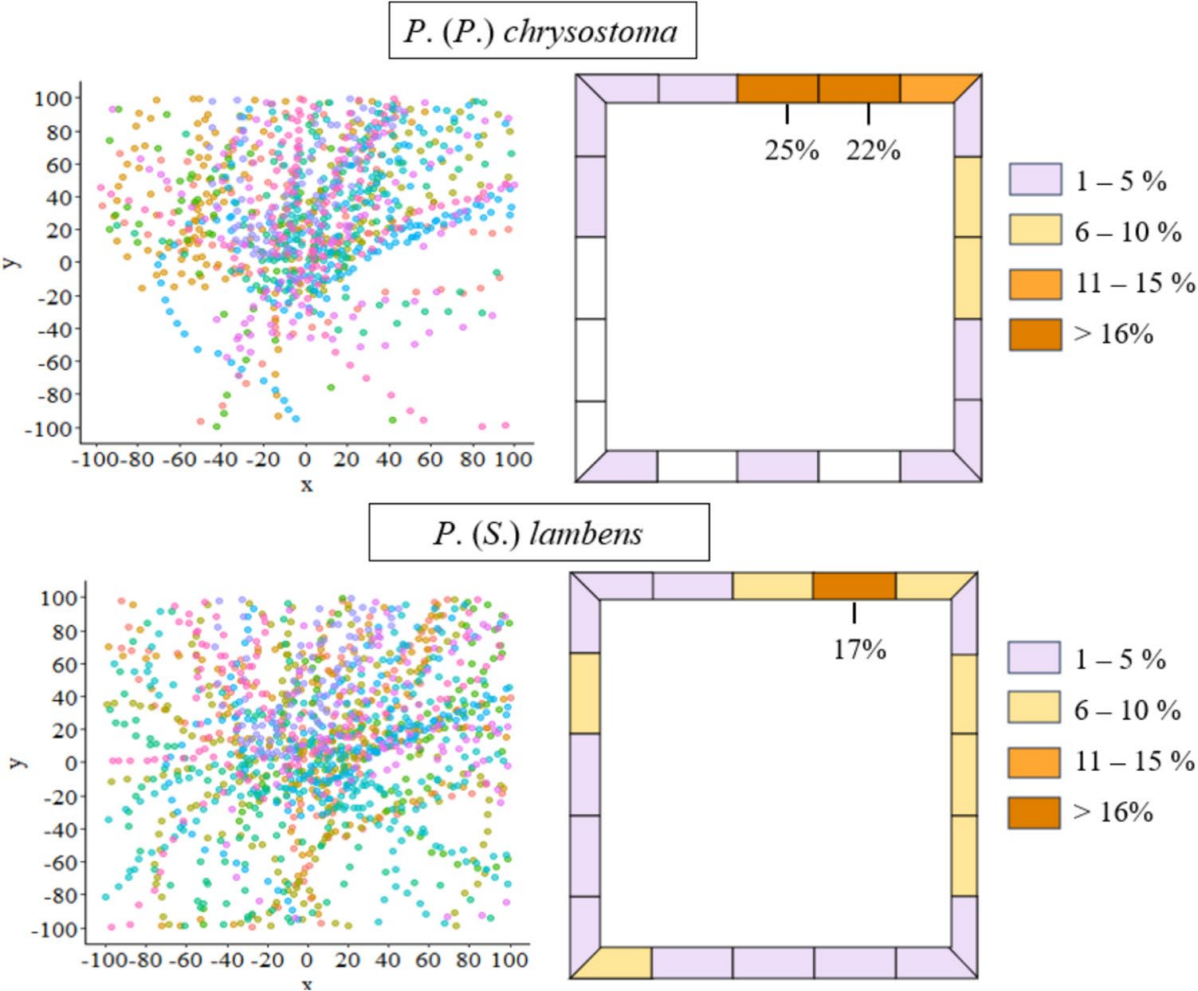
Orientation of larval movement to the Larval Mobility Arena (LMA) edges, with preferred burial areas highlighted of a) *Peckia* (*P.*) *chrysostoma* larvae and b) *Peckia* (*S.*) *lambens* larvae in each quadrant. Each colour represents a group of 10 larvae per replicate

We observed an oriented mobility. *Peckia* (*P.) chrysostoma* exhibited a predominant movement orientation towards two specific areas of the LMA, with 25% and 22% of the tested larvae moving in the same direction, suggesting a non-random pattern of dispersal. In contrast, *P.* (*S.*) *lambens* did not display a directional pattern, with the larvae moving across all sections of the LMA, but 17% of the larvae moved to the same location (Fig. 2).

The time spent in the LMA until they found the vermiculite varied for both species, with *P.* (*S.*) *lambens* staying longer in the arena (χ^2^ = 54.71, df = 1, *P* < 0.001). Other variables also showed differences, with the average speed (χ^2^ = 9.2, df = 1, *P* = 0.002) and the mean total distance travelled (χ^2^ = 3.89, df = 1, *P* = 0.04) being the mean speed and distance travelled greater for *P.* (*P.*) *chrysostoma* than for *P.* (*S.*) *lambens*. Regarding the size of the larvae, *P.* (*P.*) *chrysostoma* had a greater length (χ^2^ = 109.95, df = 1, *P* < 0.001) and weight (χ^2^ = 149.25, df = 1, *P* < 0.001) compared to *P.* (*S.*) *lambens*.

All larvae (100%) of both species exhibited burrowing behaviour immediately upon encountering the substrate (Online Resource 1 and 2). The external area, beyond the vermiculite stripe, was not used by any specimen. Additionally, there was no difference in larval burial time (χ^2^ = 0.16, df = 1, *P* = 0.68), with *P.* (*P.*) *chrysostoma* taking 42.26 ± 33.37 seconds,  while *P.* (*S.*) *lambens* required 40.91 ± 25.52 seconds,.

**Fig. 3:**
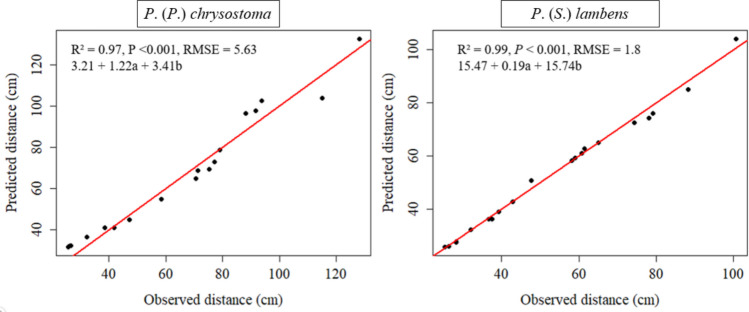
Relationship between observed and predicted dispersal distances of 3rd instar larvae at the post-feeding stage for *Peckia* (*P*.) *chrysostoma* and *Peckia* (*S*.) *lambens* using a General Linear Model (GLM)

From the data obtained in the experiments, the proposed models to predict the distances travelled for *P.* (*P.*) *chrysostoma* and *P.* (*S.*) *lambens* showed high accuracy (R^2^ > 0.95, *P* < 0.001), indicating that time and speed explained most of the variability in horizontal movement (Fig. 3).

## Discussion

Our findings provide the first empirical description of the main variables associated with post-feeding dispersal of *Peckia* species, focused in orientation, speed, and level of aggregation. So far, quantitative studies on post-feeding mobility of Sarcophagidae species have been virtually non-existent or mostly limited to the Calliphoridae species. We infer that the data have implications for forensic entomologists and behavioural ecologists.

From a forensic entomology perspective, it is reasonable to suggest that crime scene investigations should focus sampling efforts close to the cadaver, as the estimation of the min PMI is primarily based on the age of the oldest larvae. Guidelines that suggest a wider radius (> 2.0 m) for sampling Calliphoridae larvae at the crime scene may be unrealistic, given the multidirectional movement of the insects, the difficulty of finding buried pupae, and the limitations in logistics and time associated with the work of the forensic expert (Guimarães et al. 2022).

It is difficult to contextualize our findings within the current literature because most studies focus on species of the genera of Calliphoridae *Calliphora* Robineau-Desvoidy, *Chrysomya* Robineau-Desvoidy, *Cochliomyia* Townsend, *Lucilia* Robineau-Desvoidy, and *Protophormia* Townsend (Andrade et al. 2002; Roux et al. 2006; Arnott and Tuner 2008; Robinson et al. 2018). The search for entomological evidence based on Sarcophagidae may therefore be spatially limited, considering that *Peckia* larvae travel short distances, approximately 60 cm, and can bury themselves almost immediately upon finding a suitable site, usually in less than a minute. However, despite belonging to the same genus, differences exist in the overall patterns of mobility and orientation between *P.* (*P.*) *chrysostoma* and *P.* (*S*.) *lambens*.

The experimental arena for quantifying larval behaviour provided here satisfactorily allowed the registration and interpretation of the data, which leads us to suggest its use in further studies. The artificiality of the setup cannot be ignored, we chose to use a gridded acetate base to allow for videorecording, instead of soil, but the LMA was appropriate to describe the overall pattern of mobility. There is no consensus on whether larvae move slower or faster on artificial floors (e.g., concrete, tile) compared to soil (Robinson et al. 2018). Nevertheless, the LMA was designed to allow larvae to keep moving after reaching the strip of soil, in case it “decided” the distance was insufficient for a suitable place for pupation.

*Peckia* larvae moved little, but fast: it took less than a minute for *P*. (*P*.) *chrysostoma* to reach the soil, while *P*. (*S*.) *lambens* took a little longer (ca. 85 s). Despite significant differences in length and weight, *P*. (*P*.) *chrysostoma* can be twice as long and heavier as *P*. (*S*.) *lambens*, and the speed of movement was different for both species. The influence of soil humidity and undulation can be assessed in future studies, although for *Lucilia illustris* (Meigen) (Calliphoridae), the net distance travelled by larvae at the post-feeding stage did not differ between flat and undulating terrain (Freiburger et al. 2024).

Besides quantifying the total distances moved by each larva, our data can subsidize a model to predict the accuracy of the interaction between the observed and the predicted distances travelled by the larvae. These parameters can be used by the forensic experts to improve the rigor of sampling entomological evidence. For example, larvae found on cadavers in indoor environments may need to travel longer distances from the corpse until ideal pupation sites are readily available (Singh and Bala 2010). Thus, factors such as speed, presence or absence of obstacles, energy reserves, and body length can influence the horizontal distance larvae can travel (Gomes et al. 2006).

Roux et al. (2006), in a field study, observed that *Protophormia terranovae* (Robineau-Desvoidy) (Calliphoridae) exhibited irregular dispersal, similar to what was observed in this study for *P.* (*S.*) *lambens*. A slightly more oriented trajectory was observed for *P.* (*P.*) *chrysostoma* larvae, which had a higher proportion of larvae migrating to the same quadrant of the LMA. In nature, larval movement is influenced by micro-variations in factors such as soil slope, vegetation density, sunlight exposure, light direction, and humidity (Greenberg 1990; Roux et al. 2006). Biotic factors, such as the presence of predators or competing species, may also play a fundamental role in the route chosen by each larva (Faria et al. 2004; Reigada and Godoy 2005; Gomes et al. 2006).

A recent study by MacTaggart and collaborators (2025) brought interesting innovations to the methodology for describing post-larval dispersal movement, using an experimental arena known as a servosphere (Syntech TrackSphere LC-300), which allows studying insect locomotion in any direction and recording behavioural changes based on external stimuli. The authors demonstrated that the dispersal speed of larvae of the Calliphoridae species, *P. terraenovae* and *Calliphora vicina* Robineau-Desvoidy gradually decreases on solid surfaces and confirmed a negative phototropic relationship, with significantly greater displacement in the absence of light.

The LMA proposed here can incorporate several variables, such as temperature, slope, soil humidity, photophase, among others. Its simplicity, low cost, and easy assembly are additional advantages. Variations in larval mobility in relation to photophase can be quantified using the LMA, especially because the duration of light defines several aspects of the reproduction and behaviour of necrophagous flies (Ferreira et al. 2024).

The choice of the site of pupation in the vermiculite strip did not seem to be influenced by the first movers, which shows larvae wandering in a non-oriented route in the LMA. Interestingly, almost 50% of *P.* (*P.*) *chrysostoma* larvae pupated close to each other, aggregated in two of 25 quadrants, which does not discard the possibility of pheromone-mediated behaviour.

Regarding the time taken for the larvae until complete burial, both species exhibited a remarkably similar time (ca. 21 s), indicating that vermiculite was an adequate substrate and that the 5-cm depth was sufficient for complete burial. The complete burial protects the larvae from predation and allows a safe environment to undergo extreme modifications through the pupal stage until adulthood (Balme et al. 2012).

Burial depth must be superficial and in a soft soil, to allow breathing and the vertical ascension of the adult, so that the soil type can influence the duration of the pupal stage, with consequences for the estimation of min PMI. To illustrate that, the pupal stage of *Calliphora terraenovae* Macquart (Calliphoridae) was longer in clay soils compared to sandy soils (Moore et al. 2024). Pupae have been used with increasing frequency in forensic entomology as indicators of more advanced stages of the insect (Brown et al. 2015). Unfortunately, direct comparisons of our results with other flesh fly species are impossible due to the scarcity of research on Sarcophagidae.

Dry environments can increase larval mortality due to dehydration (Byrd and Tomberlin 2019; George et al. 2013), and these conditions can also directly influence larval development and pupation rates. Soil composition, particularly compaction and granulometry, can affect the larvae's ability to move and the effort required for burial. Compact soils may present a physical barrier, limiting burial depth, while looser soils facilitate digging and the pupation process (Balme et al. 2012).

However, the evidence found in this study reveals that once larvae of *Peckia* find an ideal location for pupation, they immediately bury themselves. For FE, finding pupae can provide information consistent with a longer period of cadaver colonization, and shallow excavation may be sufficient for sampling these species at the death scene. The age of empty puparia of *Phormia regina* (Meigen) (Calliphoridae) collected in the superficial soil close to a cadaver validated the estimation of min PMI in a case in Poland (Bajerlein et al. 2018). The search for pupae by forensic experts should be encouraged, as it can provide robust data for estimating the min PMI based on intrapuparial development (Dias et al. 2024).

Given the increasing relevance of pupae as entomological evidence, our findings on dispersal and burial provide insights into behavioural patterns relevant for understanding the life history of species, the decomposition dynamics in the necrobiome, and the necessity of incorporating data on insect behaviour in forensic entomology.

## Supplementary Information

Below is the link to the electronic supplementary material.

ESM 1 Record of larval dispersal of *Peckia (Peckia) chrysostoma* (Sarcophagidae) in the larval mobility arena, conducted under controlled laboratory conditions. (MP4 28.1 MB)

ESM 2 Record of larval dispersal of *Peckia (Sarcodexia) lambens* (Sarcophagidae) in the larval mobility arena, conducted under controlled laboratory conditions. (MP4 37.5 MB)

## Data Availability

All data generated or analysed during this study are included in this published article.
